# A Hybrid Approach Toward COVID-19 Pandemic Modeling in Saudi Arabia Using the Modified Susceptible-Exposed-Infectious-Recovered Model and Open Data Sources

**DOI:** 10.7759/cureus.20279

**Published:** 2021-12-08

**Authors:** Naim Ahmad, Ayman Qahmash

**Affiliations:** 1 Information Systems, King Khalid University, Abha, SAU

**Keywords:** coronavirus disease 2019 (covid-19), control measures, oxford covid-19 government response tracker (oxcgrt), saudi arabia, basic reproduction number, threshold herd immunity, infectiousness of exposed and infectious compartments, susceptible-exposed-infectious-recovered (seir) model, compartment epidemiology model

## Abstract

The coronavirus disease 2019 (COVID-19) pandemic has caused the world to operate uncharacteristically for almost the last two years. Governments across the globe have taken different control measures to eradicate it. The Oxford COVID-19 Government Response Tracker (OxCGRT) provides open access data for different countries on 20 control measures, including numerous aggregated indices. This paper employs the modified Susceptible-Exposed-Infectious-Recovered (SEIR) epidemiology model to study the COVID-19 pandemic in Saudi Arabia. The modification has been achieved by including control measures and the infectiousness of exposed compartment. A hybrid approach has been used to estimate and incorporate control measures. Initially, a composite control measure has been derived from OxCGRT data to make an attempt to fit the COVID-19 pattern in Saudi Arabia. The derived model has proven to be satisfactory through statistical tests. Nonetheless, the model patterns do not resemble the reported patterns more closely. Hence, a second heuristic approach has been utilized to devise effective control measures from the reported pattern of COVID-19 from the Saudi government agency. A satisfactory model was derived utilizing this approach with successful validation through statistical tests. Also, the model patterns more closely resemble the reported patterns of COVID-19 cases. This hybrid approach proves more robust and ensures the validity of model parameters better. The R naught (R_0_) value with the current control measures has varied from 0.515 to 1.892, with a mean value of 1.119, and is presently less than 1. The threshold herd immunity, in the absence of any control measure, is estimated to be 47.12% with an R_0 _value of 1.89 and would end up infecting 76.32% of the population. The scenario analysis with gradual partial and complete relaxations up to December 31, 2021, shows that the peaks are likely to occur in 2022; therefore, Saudi Arabia must continue to inoculate its population to eradicate COVID-19.

## Introduction

The primary instance of coronavirus disease 2019 (COVID-19) in Saudi Arabia was accounted for on March 2, 2020, and as of January 24, 2021, there is an aggregate of 366,371 confirmed cases, out of which 357,939 people have recuperated, 6,352 succumbed to disease, and 2,080 remains to be active. The COVID-19 pandemic has spread from the city of Wuhan in China in late December 2020. It mostly causes the normal cold and could cause severe acute respiratory syndrome and may develop into deadly pneumonia [1]. It transmits through direct and indirect contact, the fecal-oral route, and respiratory aerosolized droplets [2,3].

The World Health Organization (WHO) recommended physical distancing, wearing masks, washing hands with soap, and cleaning hands with alcohol-based hand rubs as possible solutions to restrict the pandemic in the absence of vaccines in the beginning. The governments had taken numerous measures to control the COVID-19 pandemic. The Oxford COVID-19 Government Response Tracker (OxCGRT) provides open access data for different countries on 20 control measures [4]. These measures are broadly classified into four categories, namely, containment and closure, economic response, health systems, and miscellaneous, consisting of eight, four, seven, and one measures, respectively. Despite the multipronged response, these measures have led to the shrinking of economies, and governments are on the lookout for opening up economic activities. Compartment epidemiology models are generally used to identify the patterns of these infectious diseases. This work attempts to utilize such a compartment epidemiology model to identify the patterns of the COVID-19 epidemic in Saudi Arabia.

The Susceptible-Exposed-Infectious-Recovered (SEIR) model is an augmented version of the original compartment epidemiology model Susceptible-Infected-Removed (SIR) [5]. For the study of the COVID-19 epidemic, numerous revised SEIR models are used that include different interventions such as social distancing, testing, public responses, and mobility restrictions [2,6-9]. The introduction of more variables brings in more complexity. Hubbs (2020) [10] introduced a single constant parameter for social distancing that was modified as the variable social distancing parameter by De Falco et al. (2020) [11]. As He et al. (2020) [12] identified the infectiousness of the exposed compartment, Dur-e-Ahmad and Imran (2020) [6] introduce different parameters to represent the infectiousness of this and infectious compartments. Similarly, a new parameter to represent the testing intervention was introduced by Berger, Herkenhoff, and Mongey (2020) [7].

This paper attempts to introduce a single parameter to represent multiple control interventions as explained in the next section. Further, the control measure is estimated through multiple approaches. It also incorporates the infectiousness of exposed and infectious compartments. Therefore, this study proposes a modified SEIR model with three additional parameters. Firstly, the model has been fitted with the composite control measure derived from the OxCGRT dataset. Secondly, a heuristic approach has been adopted to devise the effective measures from the reported COVID-19 pattern. Adoption of this hybrid approach to arrive at model parameters brings in more robustness to the results. The implementation of the modified SEIR model is done in Python language, and the results are corroborated through statistical analysis. Additionally, a scenario analysis is carried out to calculate threshold herd immunity and predict the COVID-19 pattern for partial and complete relaxations.

## Materials and methods

There are four compartments in the original SEIR model to classify society facing the epidemic: susceptible S(t), exposed E(t), infectious I(t), and recovered R(t). S(t) shows the portion that is susceptible to disease but not contracted yet at any time (t). The E(t) class is exposed to the disease but presymptomatic. I(t) individuals are infected with symptoms of coronavirus such as fever, cough, and fatigue. Finally, F(t) population has either recuperated or deceased. The mathematical formulations are presented through the following differential equations.

$\frac{dS}{dt}=-\beta.I.\frac{S}{N}$ (1) 

$\frac{dE}{dt}=\beta.I.\frac{S}{N}-\alpha . E$ (2) 

$\frac{dI}{dt}=\alpha . E-\gamma .I$ (3) 

$\frac{dR}{dt}=\gamma .I$ (4) 

where α is the inverse of central measures of incubation period, β is the average contact rate, and γ is the inverse of central measures of infectious period.

The total population (N = S(t)+E(t)+I(t)+R(t)) is assumed to be constant throughout the study interval. Similarly, it is assumed that recuperated population gains immunity from coronavirus throughout the study interval. Further, an index R naught (R0) = β/γ (basic reproduction number) is estimated, which shows the number of susceptible persons that an infectious person can infect. A value of R0 below 1 signifies that the pandemic is expected to cease.

For the modification of the SEIR model to represent interventions, studies have incorporated numerous parameters [7,10,11,13]. This work attempts to employ a single control parameter (σ) to represent the composite of 20 control measures present in the OxCGRT. The composition method has been explained in the next section. Similarly, a heuristic approach has been introduced to deduce the effective control measure from the reported COVID-19 pattern to represent σ.

Further, as the infectiousness of the exposed compartment is established in the literature [6,12], this study also has used parameters η1 and η2 to signify the relative infectiousness of exposed and infectious compartments correspondingly. These three additional parameters have modified SEIR model equations (1) and (2), and their associated changes are shown in equations (5) and (6). The last two equations (3) and (4) remain unaffected.

$\frac{dS}{dt}=-\beta .\sigma .\left ( \eta_{1} .E+\eta _{2}.I\right ).\frac{S}{N}$ (5) 

$\frac{dE}{dt}=\beta .\sigma .\left ( \eta_{1} .E+\eta _{2}.I\right ).\frac{S}{N}-\alpha .E$ (6) 

Similarly, the expression for basic reproduction number (R0) also changes. R0 is defined as “the expected number of secondary cases produced by a single (typical) infection in a completely susceptible population” [14]. A next-generation matrix is used to arrive at the mathematical equation of R0 [15]. With the help of equations (5) and (3), equations F and V are characterized to show the rate of new infections and the rate of transfer in and out of exposed and infectious populations as shown in equations (7) and (8).

$F=\begin{bmatrix} \beta .\sigma .\eta _{1} & \beta .\sigma .\eta _{2}\\ 0 & 0 \end{bmatrix}$ (7) 

$F=\begin{bmatrix} \alpha & 0\\ -\alpha & \gamma \end{bmatrix}$ (8) 

Further, the next-generation matrix is characterized as G = FV-1. The largest eigenvalue of G (the spectral radius) provides R0 as given below in equation (9).

$R_{0}=\beta .\sigma .\left ( \frac{\eta _{1}}{\alpha }+\frac{\eta _{2}}{\gamma } \right )$ (9)

Model parameters and values

There are a total of six parameters in the modified SEIR model: α, β, γ, η1, η2, and σ.

The incubation period (α) is the time period that changes an exposed individual to an infectious individual with symptoms. This time period varies from two to 14 days [16]. In the case of Saudi Arabia, the median incubation period is estimated to be six days [17]. The incubation rate is the reciprocal of this time period.

The contact ratio (β) is calculated using optimization techniques [9].

The infectious period (γ) is the time period after that infectious person either recuperates or passes away. The value of this time period varied largely in the literature, such as between 0 and 10 days [18], three and seven days [19], and 2.9 days [20]. The recovery rate is the reciprocal of this time period. The value of γ is chosen within the acceptable interval that fits the model well.

The values of the infectiousness of exposed and infectious classes (η1 and η2) are kept between 0.4 and 1 and estimated using optimization techniques [6]. In this study, the value of η2 is kept more than that of η1, assuming that the infectious individual has more likelihood of infecting a susceptible individual than an exposed individual. Further, the value of η2 is set near 1, and the value of η1 is estimated through simulation and optimization of the model.

**Figure 1:**
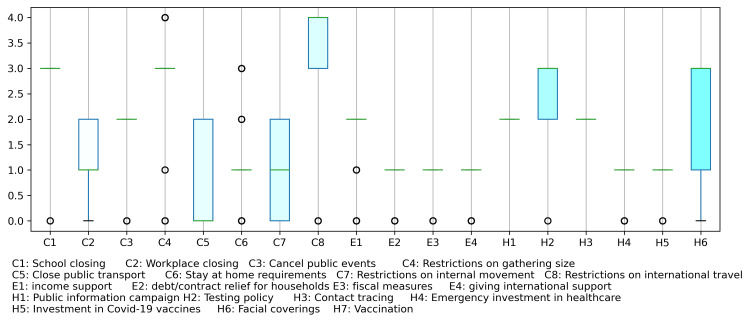
Boxplots of OxCGRT control measures for Saudi Arabia

For control measure (σ), the OxCGRT provides open access data for different countries on 20 control measures [4] (Table 1). Most of these control parameters are measured on ordinal scales and some on numeric scales. Parameters with numeric scales such as E3, E4, H4, and H5 have been marked with 1 for the presence of some values, else 0, whereas M1 is textual data and had no values for Saudi Arabia. Further, the H7 measure had no values. The boxplots of the remaining 18 control measures are shown in Figure 1.

**Table 1:** Different control measures provided by the OxCGRT

Containment and closure	Economic response	Health systems	Miscellaneous
C1: School closing	E1: Income support	H1: Public information campaign	M1: Other responses
C2: Workplace closing	E2: Debt/contract relief for households	H2: Testing policy	
C3: Cancel public events	E3: Fiscal measures	H3: Contact tracing	
C4: Restrictions on gathering size	E4: Giving international support	H4: Emergency investment in healthcare	
C5: Close public transport		H5: Investment in COVID-19 vaccines	
C6: Stay at home requirements		H6: Facial coverings	
C7: Restrictions on internal movement		H7: Vaccination	
C8: Restrictions on international travel			

**Figure 2:**
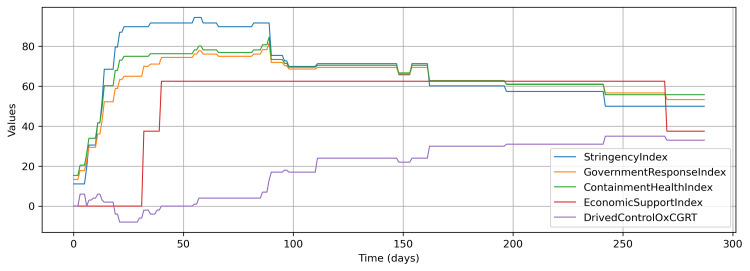
Different indices provided by the OxCGRT and derived control from OxCGRT control measures

**Figure 3:**
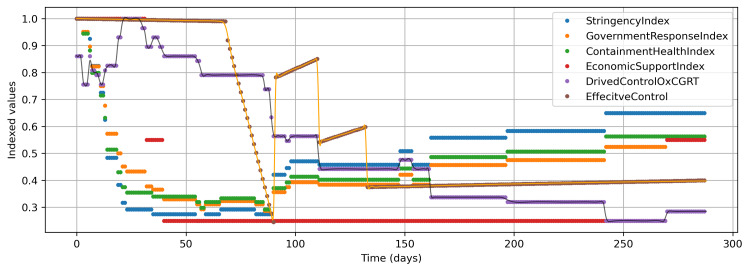
Various indices estimated from OxCGRT aggregated indices, derived control measure, and effective control measure

The OxCGRT dataset also provides some aggregated indices between 0 and 100, such as the overall government response index, stringency index, containment and health index, and economic support index (Figure 2). All these indices are aggregated with simple addition on selected control measures. Therefore, for a composite control measure for this study, a weighted aggregation approach was adopted as follows. Firstly, a weighted sum was computed by multiplying the control measure values with weights of ±1, ±2, or ±3 based on their correlation values up to ±1/3, ±2/3, or ±1, with daily cumulative confirmed cases (Figure 2). Secondly, an index was derived from this weighted sum as follows: offset with the lowest value to keep values positive, scale it to the maximum value of 0.7, and subtract values from 1. Therefore, finally, an index of 0.3 shows the maximum intensity of the control measure, and 1 represents the complete absence of any measure. Theoretically, index 0, the perfect isolation to stop any transmission of the virus, is practically unachievable. Further, the value for the maximum intensity of the control measure was varied in accordance with the second approach, and the optimal value happened to be 0.25 (Figure 3). Figure 3 also shows the indices obtained from other aggregated measures in the OxCGRT dataset in a similar fashion, and the pattern of none of these indices is similar to the derived index.

**Figure 4:**
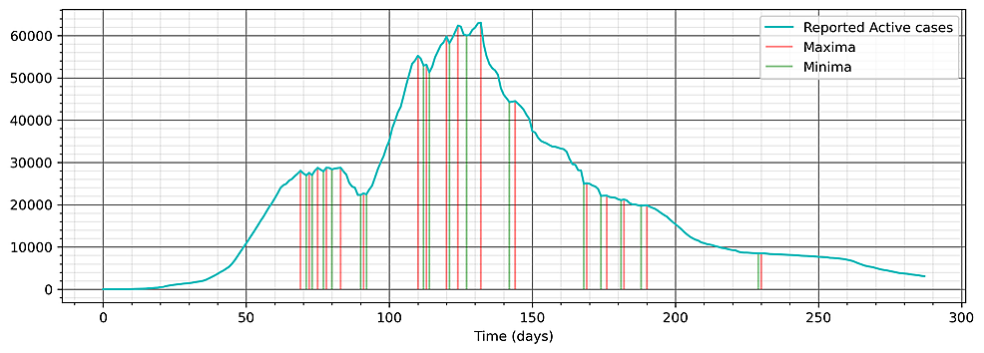
Active cases with points of maxima and minima

In the second heuristic approach, an effective control measure was devised from the reported COVID-19 pattern as shown in Figure 3. The process for the development of the effective control measure was as follows. Firstly, the reported active cases were plotted, and all the maxima and minima points were identified with python routines of argrelmax and argrelmin from scipy.signal package (Figure 4). Thereafter, appropriate points of control measures were identified, and effective control measure values were chosen based on the pattern changes and in conjunction with the model fitting. The arrived pattern in the initial stage resembles the pattern proposed in the study of Ahmad [13]. Specifically, day 90 represents the toughest controls during the Eid 2020 vacation time, wherein there was complete lockdown in Saudi Arabia; this can also be seen from other aggregated indices provided by the OxCGRT (Figure 3). The period between day 69 and day 133 had more variations in control measures. Thereafter, they have been almost the same with very slight gradual relaxation as can be seen in Figure 3 and Figure 4.

## Results

The simulation of the modified SEIR model represented by ordinary differential equations (5, 6, 3, and 4) was done in the Python language [21] using Jupyter Notebook [22]. The function solve_ivp of scipy.integrate sub-package was employed for solving equations. For the optimization purpose, the minimize function of the scipy.optimize sub-package was employed. The difference between the model and the reported values for total cases was minimized to calculate β and η1. The values of the parameters α, γ, and η2 for both approaches were as specified previously. The values of σ for both approaches have already been defined previously. Further, σ values were smoothed out employing the UnivariateSpline function with a smoothing factor of 0.01 (Figure 3). The initial conditions for solving the ordinary differential equations are as follows: I(0) = 1 (cases on the first day), E(0) = 150 (individuals exposed on the first day from index case), and R(0) = 0 (recovered on the first day). The value of E(0) has been taken high to fit the first localized peak and is quite possible as initially no measures are taken and the first case could infect a large number of individuals at multiple places. The aggregate population of Saudi Arabia is taken as 34,813,867. Due to the non-normal nature of data, Levene statistical tests are carried out to validate the model results against the reported epidemic data from the Saudi Ministry of Health [23].

**Figure 5:**
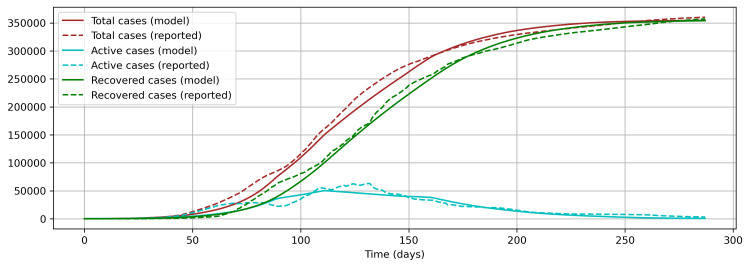
Model fitting with OxCGRT derived composite control measure index

**Figure 6:**
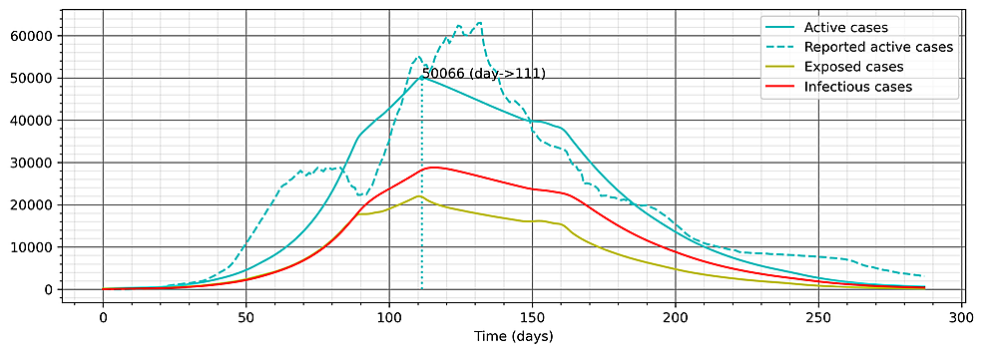
Patterns of active cases in the model and the reported data for OxCGRT derived composite control measure index

The resultant model using the OxCGRT derived composite control index identified the values of β and η1 to be 0.1868 and 0.5989, respectively, by fitting the values of cumulative total cases against the reported cumulative total cases up to December 14, 2020 (Figure 5). The Levene test (statistic = 1.0479, p-value = 0.3061) shows that there is no significant difference between the model values and the reported values. Similarly, there is no significant difference for cumulative recovered cases (Levene test’s statistic = 1.0262, p-value = 0.3112) and cumulative active cases (Levene test’s statistic = 0.1261, p-value = 0. 7225). Nonetheless, the pattern of active cases from the model and the reported cases does not match very closely (Figure 6). The second approach was sought to overcome this problem and strengthen the results further.

**Figure 7:**
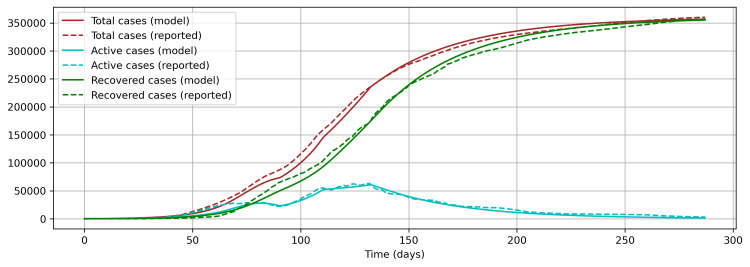
Model fitting with effective control measure devised from the reported pattern of COVID-19 cases

**Figure 8:**
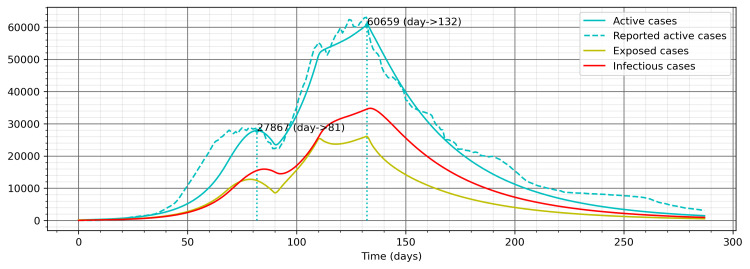
Patterns of active cases in the model and the reported data for effective control measure devised from the reported pattern of COVID-19 cases

The results from the second approach using effective control measure devised from the reported pattern of COVID-19 cases identified the values of β and η1 to be 0.1672 and 0.6108, respectively, in a similar process as in the first approach (Figure 7), and a very significant model was derived, as all the three publicly reported data, namely, total cases (Levene test’s statistic = 0.6671, p-value = 0.4142), recovered cases (Levene test’s statistic = 1.5129, p-value = 0.2189), and active cases (Levene test’s statistic = 8.2864, p-value = 0.9977), are not significantly different from the respective model values (Figure 7). Additionally, there is a closer fitness in comparison with the previous approach between the patterns of the reported active cases and the model results (Figure 8).

## Discussion

The results from both approaches depict a significant model with the same set of assumptions and parameters’ and initial values. Further, the simulations have yielded almost similar values for η1 and slightly varied values for β. As the results of the second approach seem more superior due to the closer matching of patterns, this model is used for further calculations. The R naught (R0) value has varied between 0.515 and 1.892 with a mean value of 1.119 and is presently less than 1. As the current value of R0 is less than 1, the pandemic is expected to decline in the present state of restrictions. The model also predicts that the peak has already passed with the present control measures.

**Figure 9:**
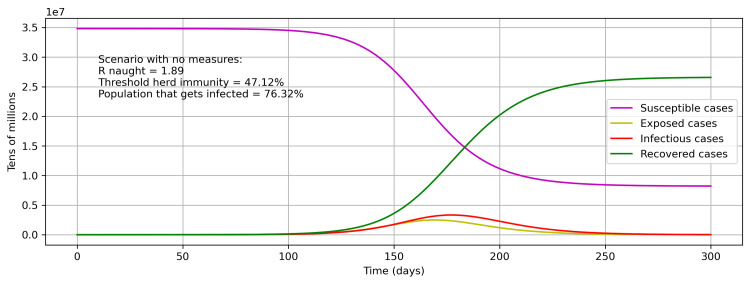
Herd immunity scenario without any control measure

The first scenario analysis has been conducted to identify the herd immunity in Saudi Arabia by removing all restrictions by making σ = 1. Thereafter, the results are recomputed from the model while keeping all the parameters’ values and initial conditions unchanged. The R0 value in this scenario is 1.89, and threshold herd immunity (1-1/R0) will be achieved at 47.12% (Figure 9). The results from this scenario show that by the end of the pandemic, 76.32% of the population would get infected. These numbers are better than the previous study [13], as the general standard of well-being is being observed more rigorously and getting imbibed in the society in addition to robustness in the results due to this hybrid approach.

**Figure 10:**
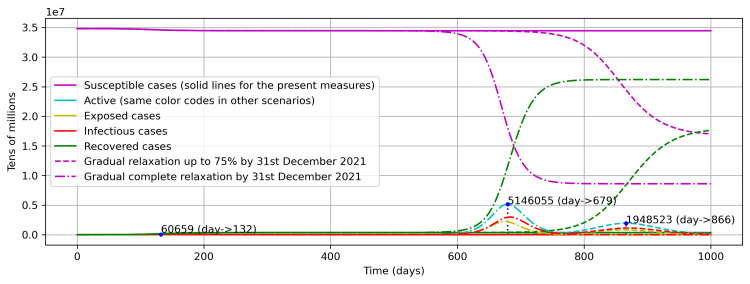
Scenario analysis for gradual relaxations by the end of 2021

Further, the second scenario was designed in line with the projected inoculation target of 70% of the population by the end of 2021 in Saudi Arabia. Hence, the effective control measures were relaxed partially up to 75% and completely by the end of 2021 in a gradual fashion. The results show that the next peak in the first case will arrive on July 16, 2022 (866 days from the first COVID-19 case) and in the second case on January 10, 2022 (679 days from the first COVID-19 case) (Figure 10). Therefore, in either case, as the peaks are lying after the year 2021, Saudi Arabia must continue its efforts to vaccinate its susceptible population to eliminate COVID-19.

## Conclusions

This study has utilized a hybrid approach for modeling the COVID-19 pandemic using a modified version of the base compartment epidemiology model of SEIR. The original model has been modified by incorporating parameters for accounting for the infectiousness of exposed and infectious compartments. Further, a single control measure has been introduced to account for multiple interventions to control the pandemic, and it was estimated through multiple approaches such as derived from the OxCGRT dataset through the composition of different control measures and heuristically devised from the pattern of reported active cases. The implementation of the modified model and optimization was carried out in a Python environment. The optimization was achieved by varying contact rates and the infectiousness of the exposed population. Both approaches have yielded a satisfactory model as indicated by statistical tests. Further, both approaches utilize the same parameters’ values and initial values that strengthen the accuracy of the results, although the model results from the second heuristic approach have provided a better fit with patterns of reported cases. The results show that the strategy adopted to ease the pandemic in Saudi Arabia has been very effective. Further, the scenario analysis shows that the gradual relaxations by the end of 2021 will cause peaks to occur in 2022. Therefore, the government must continue to inoculate susceptible populations to eradicate COVID-19. However, this study has not taken into consideration possible implications due to the different variant strains of COVID-19. Additionally, the model contains exponential functions that may cause variations in the actual reported results. Therefore, the results must be interpreted in light of the abovementioned limitations.
